# Supplementary material for: Lignin Microspheres Modified with Magnetite Nanoparticles as a Selenate Highly Porous Adsorbent
Source: Int J Mol Sci. 2022 Nov 10;23(22):13872. doi: 10.3390/ijms232213872 (PMC9696047; doi:10.3390/ijms232213872)
Supplement: Supplementary file 1 [file ijms-23-13872-s001.zip › ijms-1973357-supplementary.pdf]

International Journal of *Molecular Sciences*

Supplementary Material

Lignin Microspheres Modified with Magnetite Nanoparticles as a Selenate Highly Porous Adsorbent

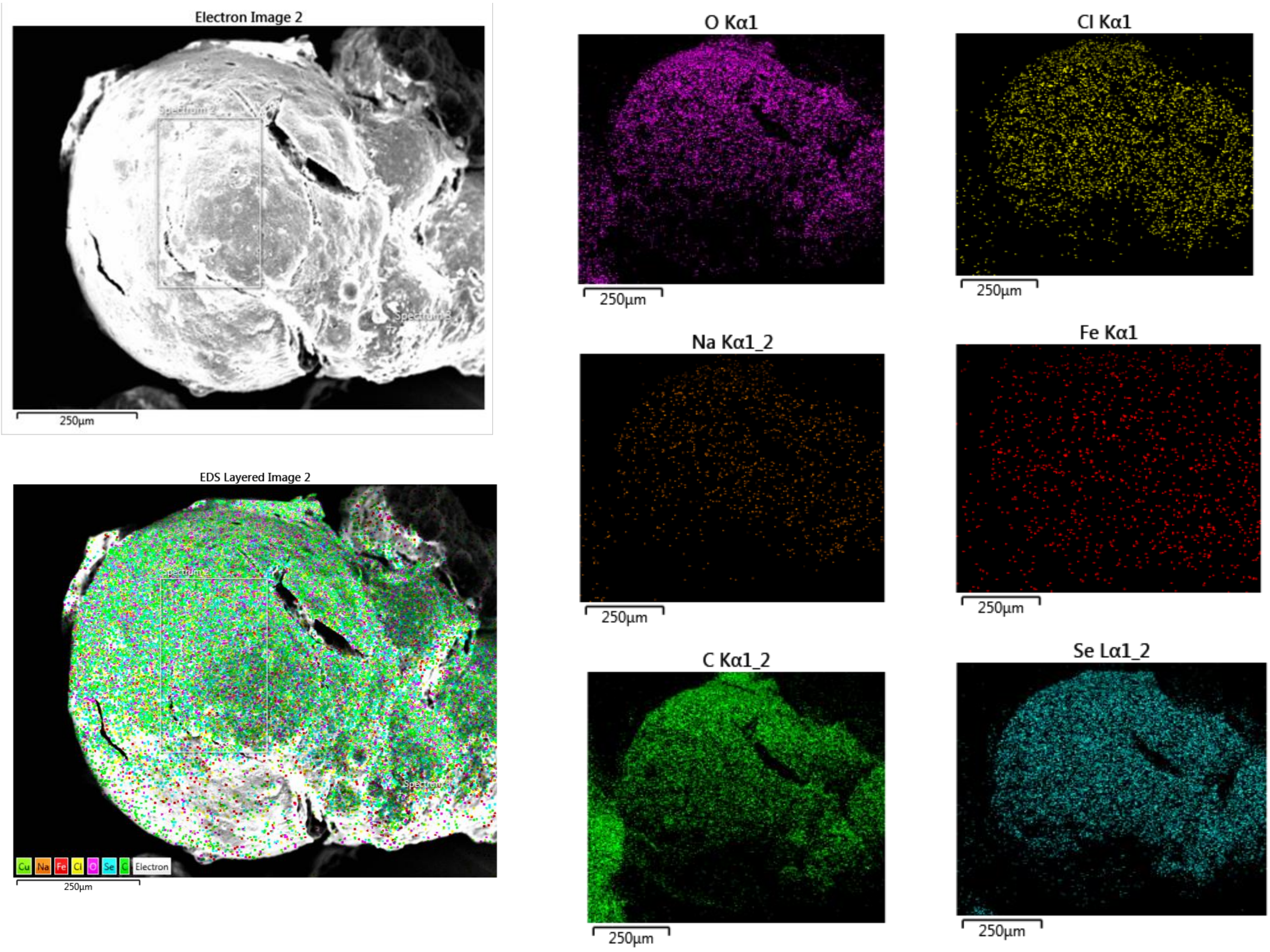

Figure S1. Map of element distribution of the A-LMS Fe_3O_4/Se adsorbent (spectrum 2).

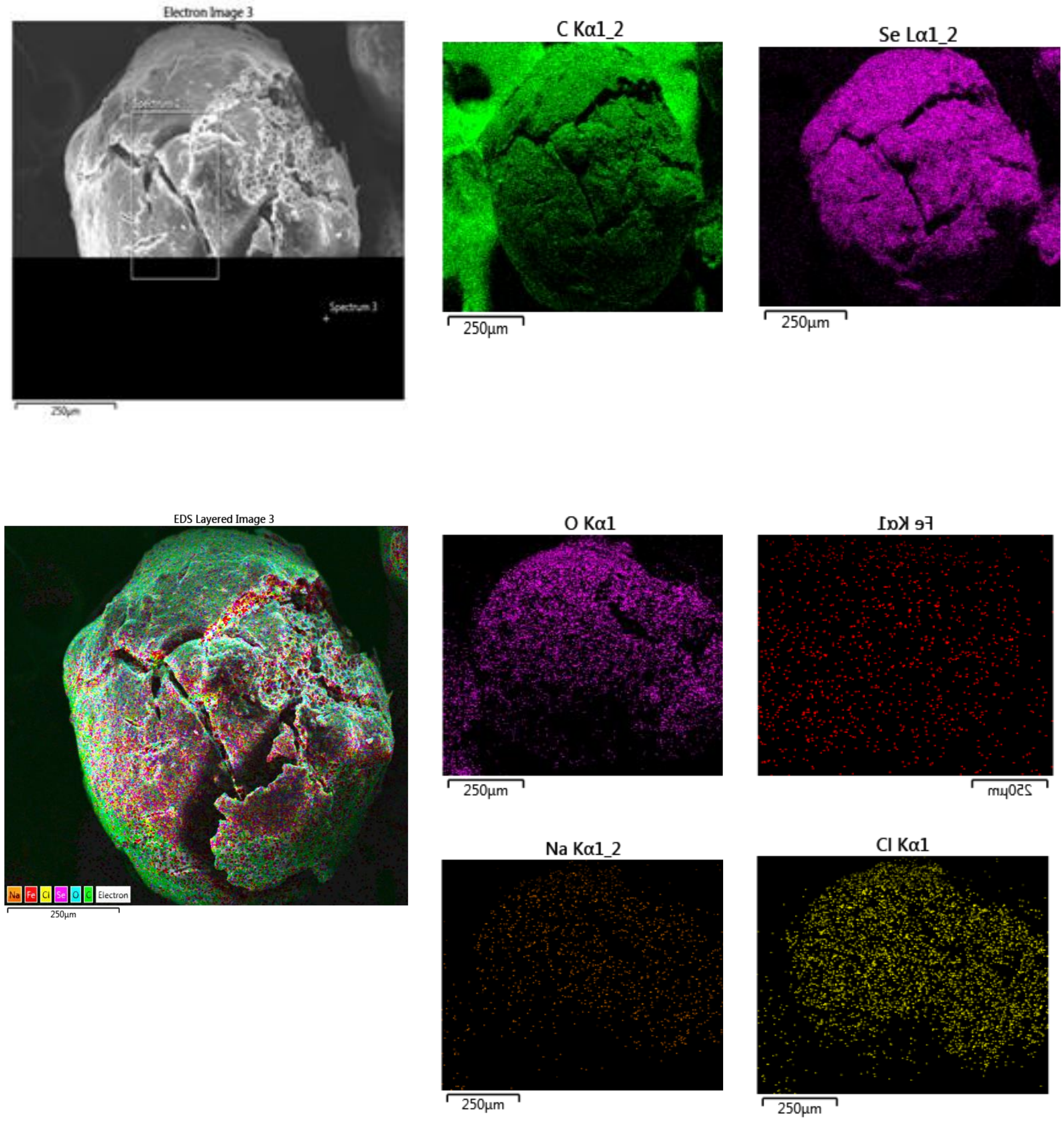

Figure S2. Map of element distribution of the A-LMS Fe_3O_4/Se adsorbent (spectrum 3).

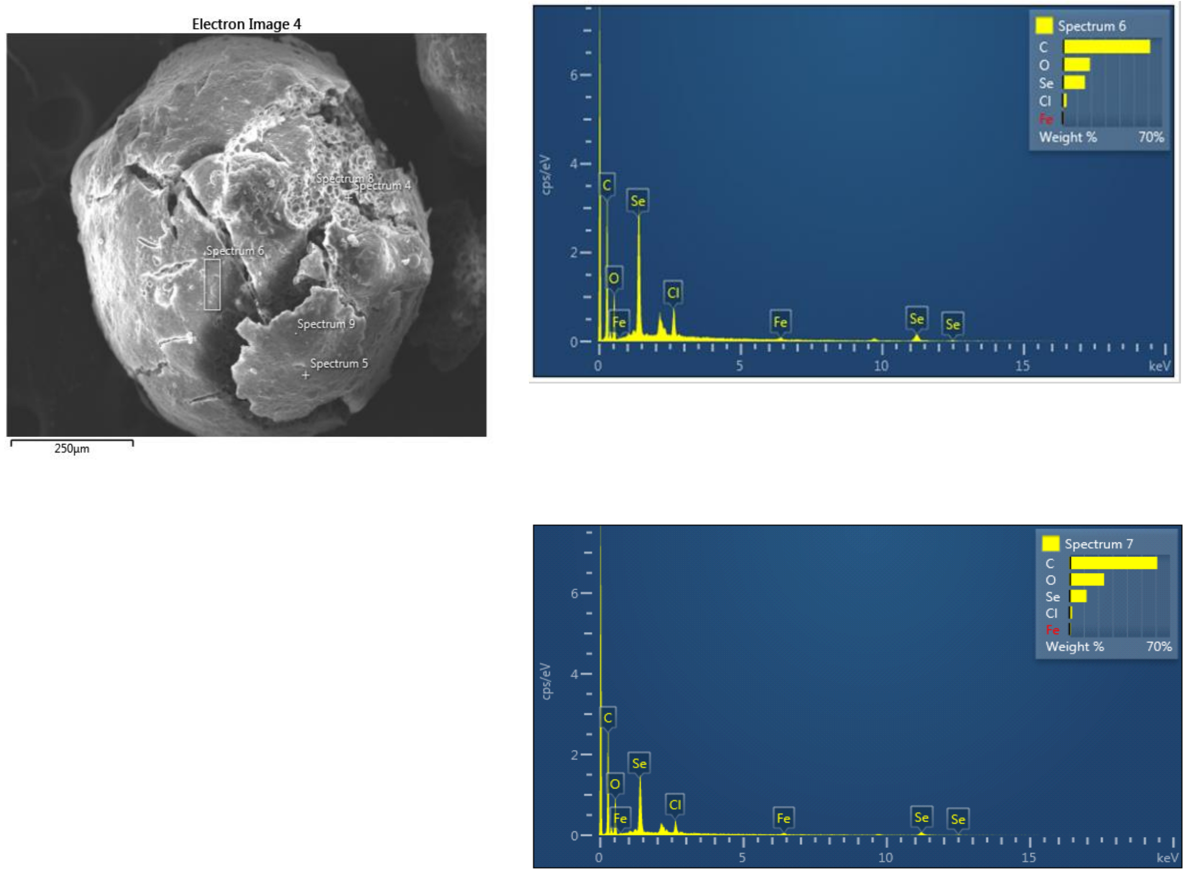

Figure S3. EDS image of the A-LMS Fe_3O_4/Se adsorbent (spectrum 6 and 7).
